# Decoding large language models for radiology: strategies for fine-tuning and prompt engineering

**DOI:** 10.1093/radadv/umaf024

**Published:** 2025-07-28

**Authors:** Sanaz Vahdati, Elham Mahmoudi, Ali Ganjizadeh, Chiehju Chao, Bradley J Erickson

**Affiliations:** Artificial Intelligence Laboratory, Department of Radiology, Mayo Clinic, Rochester, MN 55905, United States; Artificial Intelligence Laboratory, Department of Radiology, Mayo Clinic, Rochester, MN 55905, United States; Artificial Intelligence Laboratory, Department of Radiology, Mayo Clinic, Rochester, MN 55905, United States; Department of Cardiology, Mayo Clinic, Rochester, MN 55905, United States; Artificial Intelligence Laboratory, Department of Radiology, Mayo Clinic, Rochester, MN 55905, United States

**Keywords:** large language models, radiology report, fine-tuning, prompt engineering

## Abstract

The advances in large language models (LLMs) have demonstrated sophisticated potential for automating complex tasks within the radiology workflow. From radiology report generation and report summarization to data collection for research trials, these models have proven to be powerful tools. However, optimal implementation of these models requires careful adaptation to the specialized medical domain. In addition, these models tend to generate information that is not truthful or factual, which can adversely affect patient care and clinical decisions. Strategies such as fine-tuning and prompt optimization have been shown to be impactful in eliminating these errors. Although these models undergo rapid updates and improvements, understanding the principles of prompt engineering and fine-tuning provides a foundation for evaluating and maintaining the performance of any LLM deployment. The current article aims to review the recent advancements in radiology using fine-tuning and prompt optimization to leverage LLMs’ capabilities. It delves into various techniques within each strategy, their advantages and limitations, and presents a framework to facilitate the practical integration of LLMs into radiology settings.

**Abbreviations**

LLM = large language mode; AI = Artificial Intelligence; Q&A = Question and Answer; CoT = Chain of Thought; LoRA = Low-Rank Adaptation; QLoRA = Quantized Low-Rank Adaptation; PEFT = Parameter Efficient Fine-tuning; RLHF = Reinforcement Learning from Human Feedback; DPO = Direct Preference Optimization; GPU = Graphics Processing Unit

**Summary**

Large language models are promising tools for automating radiology workflows but are prone to hallucination and factual error. Fine-tuning and prompt optimization techniques significantly enhance their accuracy and clinical integration.

**Key Results**

- Large language models have demonstrated robust capabilities for automating diverse radiology tasks but have a tendency to hallucinate.
- Adaptation of these models to the medical domain through strategies such as fine-tuning and prompt engineering improves language model performance and mitigates hallucination and factual errors in radiology.
- Standardized evaluation methods are essential to ensure transparency and reproducibility for clinical purposes.

## Introduction

**Figure 1.**
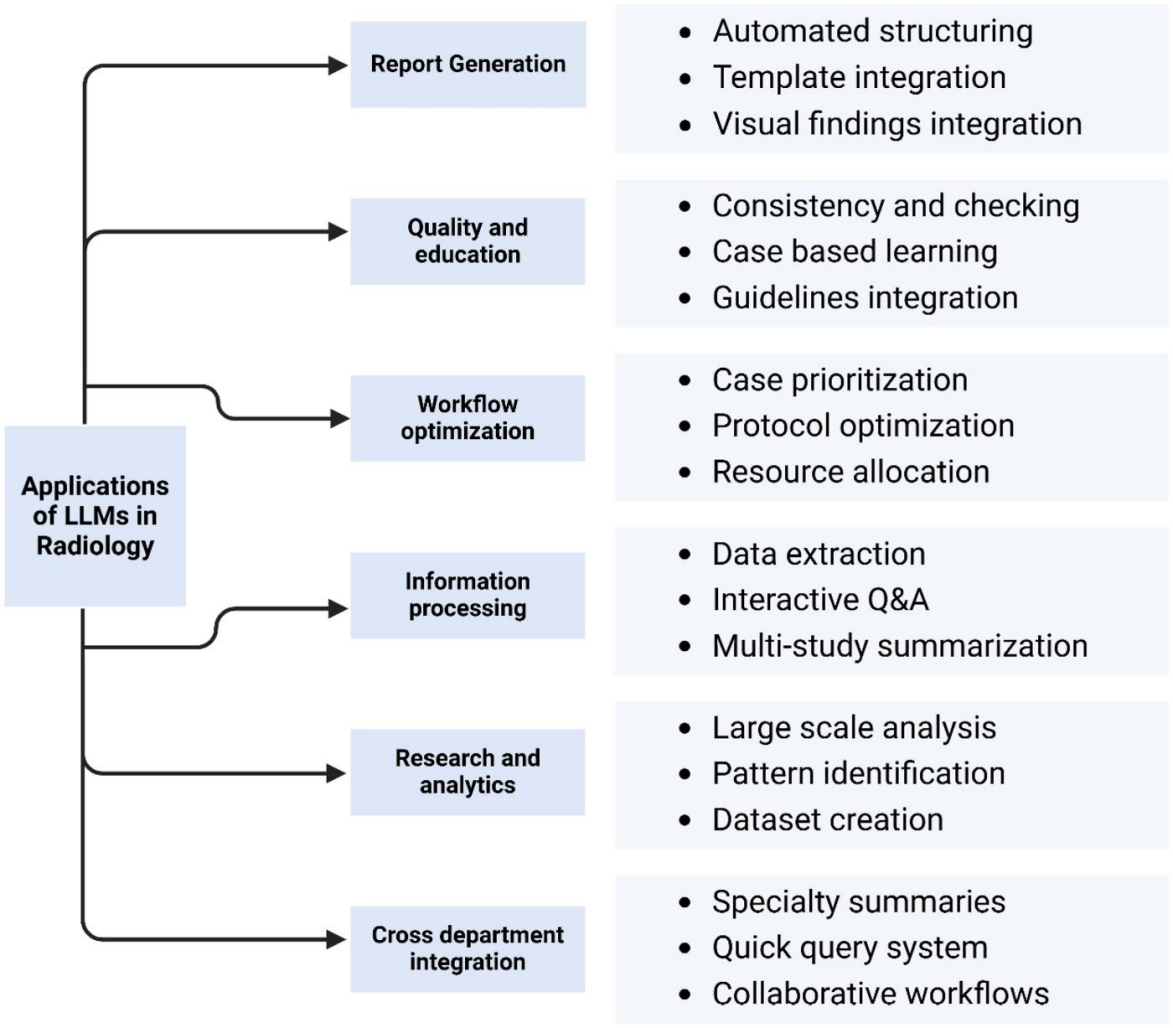
Hierarchical framework of large language models’ (LLM) applications in clinical radiology.

Radiology is undergoing a significant transformation, fueled by rapid advances in artificial intelligence (AI). Initially dominated by convolutional neural networks, the rise of large language models (LLMs) has opened up unprecedented opportunities for automating complex cognitive tasks within radiology clinical workflows. LLMs demonstrate remarkable capabilities in natural language understanding, generation, information extraction, and clinical reasoning, with promising applications in protocoling, report generation, and clinical decision support^1^^,^^2^ (Figure 1).

However, fully realizing the potential of LLMs in radiology demands careful adaptation to address their vulnerabilities, notably their tendency to “hallucinate,” or produce plausible yet inaccurate information. This potential for misinformation in the context of medical practice has raised liability concerns and calls for tailored regulatory oversight, issues that will need to be addressed for general clinical acceptance. These challenges underscore the need for specialized adaptation methods.

This review paper explores the burgeoning potential of LLMs in radiology, focusing on the strategic application of 2 key techniques, fine-tuning and prompt engineering, to enhance accuracy and improve the quality of the models’ response.^3^ Current work outlines the approach for fine-tuning with a comprehensive overview of different prompting techniques in eliciting desired responses. It provides a framework for researchers and radiologists to implement these technologies in their practice efficiently.

## Prompt engineering

A prompt is the text input that directs the model to generate output. Prompt engineering has emerged as a crucial technique in the effective utilization of LLMs for a wide range of purposes, including radiology report analysis. The LLMs are capable of providing favorable outputs for a given task through in-context learning without further training and gradient updates.^4^ The prompt’s structure and context matter significantly in LLMs’ interpretation and response generation. LLMs may generate completely different outputs in response to minorly different prompt inputs. Prompt structure not only influences the output content but also has the potential to define the format and structure of the output.^5^^,^^6^

Although the appropriate prompt varies for different models and depends on the training data, there are some general rules for aim-specific prompt optimization. An introduction to the basic prompt structure and techniques to specialize that structure for different tasks can help understand the importance of prompt engineering in text analysis.

### Prompt structure

A well-constructed prompt that is supported by most of the commonly used LLMs should be structured as a few primary conversational roles. Although OpenAI’s ChatGPT and many open-source LLMs support a common set of primary roles, including “system,” “user,” and “assistant,” Anthropic’s Claude and Google-based LLMs do not follow an explicit role-based structure. Declarative Self-improving Python (DSPy)^7^ also follows its own role system, including “prompt,” “example,” and “reflection” roles. Here, we describe the system, the user, and the assistant roles as the most used role system (Supplementary Material).

In the context of radiology report analysis, prompts may include queries or instructions to summarize or structure the report. Instruction prompting of LLMs focuses on following human instructions and enables them to be particularly adept at understanding and executing complex prompt instructions, like differentiating clinical pathologies and categorizing the severities. For example, Nguyen et al. investigated the potential of ChatGPT in providing the Breast Imaging and Reporting Data System (BIRAD) score using the American College of Radiology guideline and proposed that structured integration of established criteria improved LLM’s performance; however, further investigation is required to prevent overestimation of the severity of the cases.^8^ This is particularly important in radiology applications, where precise terminology and structured outputs are essential.

### Zero-shot prompting

Zero-shot prompting represents the most direct approach to using LLMs, where the model performs tasks without prior examples. Performance relies entirely on the model’s pretraining and the clarity of the prompt. This approach has shown promise in simpler tasks, such as classification or basic medical terminology interpretation, but struggles with complex clinical reasoning.^9^^,^^10^

Park et al. proposed a patient-centered radiology report generation. They used ChatGPT with zero-shot prompting to create a summarized report that is patient-friendly and obtains further recommendations to optimize communication with patients.^11^ Zero-shot prompting is straightforward and widely applicable but often serves as a baseline for more advanced techniques.

### Few-shot prompting

Few-shot prompting enhances the basic prompting paradigm by incorporating examples directly within the prompt structure. This approach provides the model with concrete examples of desired input-output pairs, effectively creating a mini-training set of annotated data within the prompt itself.^12^ Despite the initial belief that “language models are few-shot learners,” case studies questioned this by showing better performance for zero-shot prompting, particularly in smaller LLMs.^13^^,^^14^ It seems that larger models can benefit more from few-shot examples. Additionally, the selected examples should ideally cover different aspects of the task while maintaining consistency in format and quality. This will help the model to understand specific patterns, output formats, and domain-specific requirements; thus, carefully choosing examples that represent both typical cases and edge scenarios can improve model performance.^15^ Yan et al. explored a style-aware approach to radiology report generation by decoupling content extraction and style extraction. They used a 2-step approach to first generate radiology reports from images and then applied few-shot prompting to stylize them. Radiologist evaluations found the generated reports to be indistinguishable from individual radiologists. The authors noted that this approach, combining stylization with in-context learning, could improve flexibility and consistency and highlight the most relevant findings to a specialist’s scope of practice.^16^

### Chain-of-thought prompting

Chain-of-thought (CoT) prompting is an advanced technique that explicitly integrates intermediate reasoning steps into the prompt structure, guiding the model through a step-by-step process that culminates in a conclusion (Supplementary Material). This approach is shown to improve the model’s answers, particularly to logical and computational questions; it also makes the reasoning process transparent and verifiable by breaking down complex tasks into intermediate steps.^17^ The explicit decomposition of complex reasoning chains allows for better verification and correction of potential errors at each step of reasoning. CoT prompting may be used in a zero-shot approach or combined with few-shot examples of reasoning chains, known as “golden CoT,” for better performance.^18^

Bhayana et al. investigated the use of ChatGPT to create structured radiology reports for pancreatic ductal adenocarcinoma assessment. They reported that using ChatGPT with CoT reasoning outperformed in-context prompting, and surgeons using these reports demonstrated greater accuracy in determining lesion resectability compared to those using standard reference reports.^19^ Furthermore, CoT has shown capabilities in improving complex diagnostic reasoning and differential diagnosis development.^20^ Examples of various prompting strategies for radiology report analysis are summarized in Table 1.

**Table 1.** Examples of 3 forms of prompting techniques for data extraction from a radiology report.

**Report:** Acute nondisplaced fracture of the left parietal calvarium. There is an overlying left parietal scalp hematoma. Acute subarachnoid hemorrhage in the right frontal sulci as well as the right sylvian fissure. No evidence of an intraparenchymal hematoma. No extra-axial fluid collection. Ventricles are normal without evidence of blood products. Preserved gray-white differentiation. There is no evidence of an acute facial fracture.	
Prompt Engineering Technique	Sample Radiology Prompt
**Zero-shot Prompting**	Prompt: You are a neuroradiologist. Carefully review the provided radiology reports. Determine if there is any intracranial hemorrhage present in this report. If yes, identify the type of intracranial hemorrhage. Report: [Report] Answer: Yes, subarachnoid hemorrhage
**Few-shot Prompting**	Prompt: You are a neuroradiologist. Given these examples, carefully review the provided radiology reports. Determine if there is any intracranial hemorrhage present in this report. If yes, identify the type of intracranial hemorrhage. Example 1: Report: “…There is mild subarachnoid hemorrhage involving the anterior superior aspect of the right temporal lobe with mild extension into the parietal lobe. There are no additional areas of intracranial hemorrhage. The ventricles are normal in size, shape, and position. There is no evidence of an acute infarct. Answer: “subarachnoid hemorrhage.” Example 2: Report: “…Stable subdural hemorrhage spanning 8 mm on the right side of the falx. Scattered white matter disease. Calcification in the left choroid plexus. No significant mass effect. No fractures. Lens extractions. Scalp defect.… Answer: subdural hemorrhage. Now select the correct option in this report: [Report] Answer: Yes, subarachnoid hemorrhage
**Chain of Thought Prompting**	Prompt: You are a neuroradiologist. Given these examples, carefully review the provided radiology reports. Determine if there is any intracranial hemorrhage present in this report. If yes, identify the type of intracranial hemorrhage. Example 1: Report: “…There is mild subarachnoid hemorrhage involving the anterior superior aspect of the right temporal lobe with mild extension into the parietal lobe. There are no additional areas of intracranial hemorrhage. The ventricles are normal in size, shape, and position. There is no evidence of an acute infarct. Answer: “subarachnoid hemorrhage.” Example 2: Report: “…Stable subdural hemorrhage spanning 8 mm on the right side of the falx. Scattered white matter disease. Calcification in the left choroid plexus. No significant mass effect. No fractures. Lens extractions. Scalp defect.… Answer: subdural hemorrhage. Now select the correct option in this report: [Report] Answer: 1. Yes, subarachnoid hemorrhage. 2. The report mentions “acute subarachnoid hemorrhage.” 3. The evidence supports the presence of subarachnoid hemorrhage.

### Prompt optimization

A common approach to prompt optimization in LLMs involves iterative evaluation of model outputs to identify sources of misinterpretation and revise the prompt accordingly to reduce errors, particularly hallucinations. In radiology, this process is ideally conducted by domain experts such as radiologists whose specialized knowledge ensures alignment with clinical standards. However, when prompts are designed to simplify, summarize, or categorize information for a patient or general population, the prompt optimization may be guided by nonexpert collaborators from the target population.^21–23^ Schmidt et al. demonstrated that LLMs could detect speech recognition errors in radiology reports and enhance overall reporting accuracy through prompt refinement.^24^ The repetitive task of prompt modification and output evaluation may be guided heuristically or based on quantitative metrics until the outputs stop improving or the metric reaches a specific performance level.^25^^,^^26^ A few steps of CoT prompting can facilitate this process by elucidating the reasoning behind the model’s conclusions. Recent advances in prompt engineering have led to the development of automated pipelines for prompt optimization, reducing the manual effort required in prompt engineering while improving consistency and performance. These techniques may employ a rule-based paraphrasing of the initial prompt content based on a sample of optimized prompts or a task-specific template (eg, LangChain, PromptSource),^27–29^ they may instruct the LLM itself to generate an optimized prompt for a specific task (showcased in recent OpenAI GPT plugins), they may rely on user feedback (eg, AutoPrompt), or may incorporate prompt embedding (Supplementary Material) to apply textual gradients (inspired by gradient-descent backpropagation), for iterative prompt evaluation and optimization (eg, Microsoft’s APO).^30^

All these approaches require robust validation to guide them in an automatic optimization process. Some of the common techniques employed for textual prompt validation are summarized in Table 2.

**Table 2.** List of common textual prompt validation techniques.

Textual Prompt Validation Techniques	Description
**Human feedback loops**	Interactive user feedback to confirm or score prompts in each iteration. These feedbacks are often used in the process of reinforcement learning^66^^,^^67^.
**Scoring systems**	Value a prompt in comparison with a standard prompt content or semantic structure^23^^,^^29^.
**Evolutionary search-based techniques**	To find the best-performing prompts by evaluating a range of possibilities utilizing the same methods used in genetic algorithms^68^.
**Soft prompting techniques**	Also known as continuous prompting, replace the textual prompt inputs with embeddings generated during the model’s training. This embedded content is more flexible for gradient-based training and fine-tuning^69^.

### Programmatic prompting

These prompting approaches, although following a specific template and guidelines, still rely on manual crafting and a trial-and-error process. To overcome this limitation, a team from Stanford University developed the DSPy package—a programming framework designed to streamline the development and optimization of language model pipelines by abstracting away from manual prompt engineering. Instead of relying on hand-crafted prompt templates, which can be brittle and hard to scale, DSPy uses a compiler to generate optimized LLM invocation strategies from a program automatically. The core idea is to treat language models as abstract devices for text generation and optimize their usage in computational graphs. DSPy achieves this through key abstractions like natural language signatures, parameterized modules, which replace hand-prompting techniques, and teleprompters, which optimize modules to maximize a specified metric. Using DSPy’s compiler, LLM pipelines can be expressed as programs in Python. The DSPy compiler then optimizes these pipelines to improve quality or cost, using training inputs and a validation metric to bootstrap example traces for self-improvement. The authors found that, within minutes of compiling, a few lines of DSPy allow GPT-3.5 and llama2-13 b-chat to self-bootstrap pipelines that outperform standard few-shot prompting methods. DSPy contrasts with other libraries like LangChain and LlamaIndex by focusing on core composable operators and automatic compilation rather than prepackaged components that rely on manual prompt engineering.^7^ Gandomi et al. applied DSPy to automatic prompt optimization for detecting bilateral infiltration in radiology reports, aiding the classification of acute respiratory syndrome.^31^

The decision between different approaches and choosing the right techniques is domain- and task-specific (Table 3). However, rigorous validation and expert oversight remain essential, particularly in clinical settings where accuracy and reliability have a prominent impact on patient outcomes.

**Table 3.** Overview of prompting techniques with radiology studies mentioned.

Technique Type	Example Radiology Application	Key Teaching Point
**Zero-shot Prompting**	Radiology report generation using ChatGPT to create patient-friendly summaries with recommendations^11^	Most direct approach relying solely on pre-trained knowledge; effective for simple classification
**Few-shot Prompting**	Style-aware radiology report generation, producing outputs indistinguishable from individual radiologists^16^	Incorporates concrete input-output examples within prompt structure; careful selection covering typical cases and edge scenarios improves performance
**Chain-of-Thought (CoT) Prompting**	Structured radiology reports for pancreatic ductal adenocarcinoma improv surgeon accuracy in resectability categorization^19^	Explicitly incorporates step-by-step reasoning and intermediate conclusions; makes reasoning process transparent and verifiable
**Programmatic Prompting**	Automatic prompt optimization detecting bilateral infiltration to classify acute respiratory syndrome disease from radiology reports^31^	Abstracts away from manual prompt engineering using compiler-generated optimized LLM strategies

## Fine-tuning

Fine-tuning is a critical step in adapting LLMs for specialized tasks and extracting domain-specific insights. Although the term “fine-tuning” is sometimes used interchangeably with “prompting” in the literature, the 2 are fundamentally different, as fine-tuning changes model weights and prompting does not.^32^ Pretraining equips models with generalized knowledge by exposing them to extensive and diverse datasets. In contrast, fine-tuning narrows the model’s focus by optimizing parameters based on task-specific data.^33^ Fine-tuning enhances the model’s ability to perform specialized functions such as analyzing radiology reports or generating diagnostic impressions. To meet the specific demands of the radiology field, fine-tuning strategies have evolved to balance performance optimization with practical constraints including limited labeled data, computational resource limitations, and regulatory requirements. Fine-tuning methods can be broadly categorized into task-specific approaches (2.1, 2.2, 2.3) and alignment-focused techniques (2.4, 2.5), each serving distinct objectives in adapting LLMs for practical applications (Table 4).

**Table 4.** Overview of fine-tuning techniques with supporting radiology studies mentioned.

Fine-tuning Method	Example Use Case	Key Teaching Point
**Traditional Full Fine-tuning**	Radiology foundation model for disease diagnosis and report generation^35^	Updates all model parameters, particularly valuable for multimodal integration of text and imaging data
**Instruction Tuning**	Radiology-GPT to create an impression from the findings in text, mimicking radiologist’s workflow^38^	Structured training data as instruction-input-output triplets
**Parameter Efficient Fine-tuning (PEFT)**	QLoRA applied to Llama3-70B using 6.5 million radiology reports for impression generation^44^	Reduces trainable parameters significantly while maintaining performance
**Reinforcement Learning from Human Feedback (RLHF)**	LLM-as-a-judge mechanism with automated preference evaluation for CXR report generation^47^	Trains a model through a reward-based paradigm using human preferences
**Direct Preference Optimization (DPO)**	Fine-tuning a vision-language model for radiology report generation to avoid fabricating prior exams comparisons while maintaining clinical accuracy^49^	Directly optimizes the model based on expert preferences without a reinforcement learning loop

### Traditional full fine-tuning

This approach involves updating all parameters of a pretrained model using labeled radiology data. Although effective, it is resource-intensive and may be impractical for models with billions of parameters, such as GPT-4. Traditional fine-tuning is mostly a form of supervised learning, where the model learns from labeled examples to map inputs to outputs. In the context of fine-tuning, a pretrained model is specialized on a new task or dataset by showing examples with corresponding labels. For example, Nishio et al. fine-tuned the T5 open-source language model using the MIMIC Chest X-ray database and the Japan Medical Image Database. Their evaluation, based on both ROUGE metrics and expert radiologist assessment, confirmed that the fine-tuned models could generate clinically meaningful summaries of radiology reports.^34^ Wu et al. introduced the Radiology Foundation Model, a visually conditioned autoregressive model pretrained on a massive multimodal dataset and fine-tuned using 3 million radiologic images with high-quality language instruction and response. Their model enables the integration of natural language with 2- or 3-dimensional medical imaging and is provided as a tool for modality recognition, disease diagnosis, and report generation.^35^ Zhang et al. proposed a vision-language model, “BiomedGPT,” and reported that fine-tuning enables it to perform well on diverse biomedical datasets and imaging modalities without requiring extensive computational resources.^36^

### Instruction tuning

Instruction tuning is a specific type of supervised fine-tuning where the training data are structured to include explicit instructions along with the input and desired output. The training data are presented as instruction-input-output triplets. This approach is designed to improve the model’s ability to follow instructions and generalize to unseen tasks. This method can also incorporate CoT data to improve the model’s reasoning abilities. Singhal et al. showcased this technique with Flan-PaLM, where instruction tuning improved performance on medical question-answering benchmarks.^37^ Liu et al. introduced Radiology-GPT. Their aim was to tune the model so that it could generate an impression text given a findings text as an instruction, essentially mimicking the work of a radiologist. The underlying language model learns the relationship between findings and impressions from the dataset and, hence, starts generating impressions in a similar manner when presented with new findings.^38^

### Parameter efficient fine-tuning

Parameter efficient fine-tuning provides an effective technique by decreasing the number of parameters and the memory required for fine-tuning, yet offering performance on par with traditional full fine-tuning. It includes various methods, such as partial fine-tuning, additive fine-tuning, and reparameterized fine-tuning. Low-rank adaptation (LoRa) and LoRa derivatives are 2 subsets of reparameterized fine-tuning.^39^ In 2021, Hu et al. demonstrated that LoRA decreases trainable parameters by 10 000 times while maintaining model performance.^40^ LoRA is a method for efficiently adapting large, pretrained language models to specific tasks. Instead of modifying all the model’s parameters, LoRA freezes the pretrained weights and introduces trainable, low-rank matrices into the Transformer architecture.

By keeping the pretrained model’s weights frozen and training only the low-rank matrices, LoRA significantly reduces the number of trainable parameters, as well as memory requirements, during training. This approach is also beneficial since it maintains or improves model quality. Furthermore, the trainable matrices can be merged with the frozen weights before deployment, which means that there is no additional inference latency (ie, inference latency is the time taken by a model to process an input and produce an output).

Building on this foundation, Dettmers et al. introduced QLoRA as an efficient fine-tuning approach designed to reduce memory usage, enabling the fine-tuning of large LLMs on a single GPU (Supplementary Material). Unlike standard LoRA, which works with full-precision pretrained models, QLoRA uses a 4-bit quantized pretrained language model and significantly reduces memory requirements, making it possible to fine-tune a 65-billion parameter model on a single 48-GB GPU.^41^ Veen et al.^42^ applied QLoRA for summarizing radiology reports and progress notes, noting its potential to reduce documentation burden. Chen et al.^43^ used QLoRA to fine-tune open-source models for radiology differential diagnosis, building a large dataset from ChatGPT-labeled impression sections and showing improved diagnostic accuracy. Shi et al.^44^ fine-tuned Llama3-70B with QLoRA and traditional methods on >6.5 million deidentified radiology reports to generate impressions from findings, observing that QLoRA offered comparable performance to full fine-tuning with significantly reduced computational cost for large parameter models. Additionally, Chao et al.^45^ achieved expert-level echocardiography report summarization by applying QLoRA to fine-tune Llama-2-7B.

### Reinforcement learning from human feedback

Reinforcement learning is a type of machine learning technique in which the model is trained by a reward-based paradigm to achieve the optimal result. Designing an effective reward system is essential for guiding the model towards desired behaviors. Reinforcement learning from human feedback (RLHF) considers human preferences to align the model outputs with human standards. It progresses through supervised fine-tuning, preference sampling, reward learning, and, finally, reinforcement learning fine-tuning and optimization.

**Figure 2.**
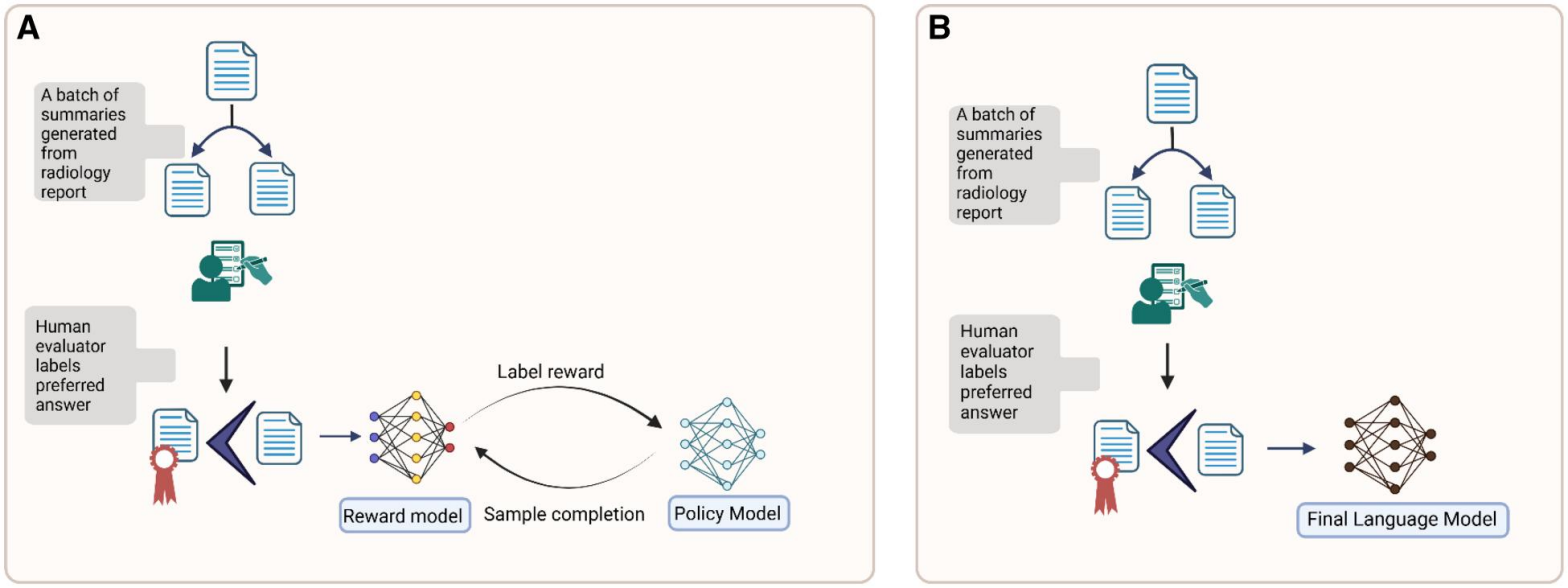
Schematic description of (A) reinforcement learning human feedback (RLHF) and (B) direct preference optimization (DPO) for summarization of a radiology report. In the first step, the radiology report is summarized by the model, and samples are collected for the same report. Then it is reviewed by a human evaluator (eg, radiologist) to define the preferred output from the model. The preferred summary is then fed to the reward model to update the relevant policy in each strategy.

This method typically starts with a pretrained model that is further fine-tuned using supervised learning on a specific dataset. The model then collects different initial outputs of the model as samples and sends a batch of pairs to a human expert to label them based on preference. In the next step, a preference loss is defined, and a reward model is trained using the preferred feedback from human experts. In the final step, which is the reinforcement learning training loop, the model undergoes fine-tuning using the updated reward model and trains a policy that optimizes the learned reward model (proximal policy optimization). This process allows the model to generate responses that are more likely to receive higher rewards, optimizing alignment with expert preferences^46^ (Figure 2). Nevertheless, the requirement for substantial feedback from radiologists has thus far hindered its adoption in the field. Ongoing efforts have focused on reducing the dependency on human experts and automating the preference evaluation of responses. Hein et al. proposed an LLM-as-a-Judge mechanism. They employed an LLM-based metric to evaluate generated chest x-ray reports. They used radiology reports written by radiologists as reference standards and created a fully automated preference dataset for fine-tuning.^47^ The application of RLHF in radiology may prove beneficial as it aligns model outputs with clinical goals.

### Direct preference optimization

Direct preference optimization (DPO) provides a computationally efficient and stable alternative to RLHF. DPO relies on a preference model to measure how well a given reward function aligns with empirical preference data without the reinforcement learning training loop. It directly optimizes the model based on expert preferences. Human experts evaluate the pair outputs and label their preference, allowing the LLM to learn and adjust its behavior. It optimizes a policy using a simple binary cross-entropy objective to obtain the optimal policy (Table 5).^48^ Banerjee et al. applied DPO to fine-tune a vision-language model for radiology report generation, targeting the suppression of hallucinated references to prior examinations. By training on GPT-4-curated preference pairs, the model learned to avoid fabricating prior comparisons without compromising clinical accuracy.^49^

**Table 5.** Comparison of features between direct preference optimization (DPO) and reinforcement learning from human feedback (RLHF).

Feature	Direct Preference Optimization (DPO)	Reinforcement Learning from Human Feedback (RLHF)
**Approach**	Supervised contrastive learning	Reinforcement learning with a reward model
**Computational Cost**	Lower	Higher (requires reward modeling and reinforcement learning updates)
**Implementation Complexity**	Simple	More complex (multistage)
**Training Stability**	More stable	It can be unstable (reward hacking, Proximal Policy Optimization instability)
**Efficiency**	Faster, requires fewer resources	Computationally expensive
**Prone to Reward Model Bias?**	No	Yes

### Fine-tuning for factual correctness

Hallucination is a phenomenon in which the model generates content that deviates from the input, contradicts previous generated outputs, or gives baseless and untruthful information. For instance, the LLM might report a large pleural effusion in the right lung when the input (text or image) shows no such finding. Factuality in the context of LLMs pertains to a model’s capacity to learn, collect, and apply accurate information. It is often defined as the likelihood that LLMs generate information that is not consistent with established facts. For example, the LLM might correctly identify a consolidation in the left lung but incorrectly describe it as consistent with pneumonia when it is actually more suggestive of a lung tumor. Information related to hallucinations might not always include factual errors. Even if the generated text deviates from the original prompt’s details, it still comes within hallucinations and isn’t always a factual problem if the content is correct.^50^ Several approaches, such as FactScore and Factcheck-GPT, have been introduced to evaluate the performance of LLMs on factual correctness.^51^^,^^52^ Enhancing factual correctness can be achieved through in-context learning by injecting updated or correct information to rewrite the false facts. Supervised fine-tuning has shown capabilities to decrease factual errors as well. Delbrouck et al. used the RadGraph dataset to fine-tune their PubMedBert model and applied a reinforcement learning reward approach to enhance the factual correctness of their model.^53^

Another important technique to improve model performance is retrieval augmented generation. With this strategy, the model will collect a corpus of information and embed it as vectors injected into the LLM. Although some studies mention this strategy as a possible substitute for fine-tuning, it is proposed that in practice, a combination of a moderately fine-tuned model that’s already radiology-adept, with retrieval augmented generation for specific facts and references, improves comprehension and relevance of the generated answer.^54^

## Applications in radiology

Advancements in LLMs hold promise for improving patient outcomes, optimizing radiology practices, and democratizing AI access in health care. Radiology workflows are often burdened by repetitive and time-intensive tasks such as summarizing findings, generating radiology reports and transforming them to structured reports, simplifying reports for patients, and selecting appropriate imaging protocols^33^ (Figure 1). In addition, automatic labeling of reports can help prioritize important clinical findings, leading to timely intervention and enhancing the quality of care. LLMs have shown the potential to automate these processes, allowing radiologists to concentrate on complicated cases.

In our current work, we delved into prompt optimization and fine-tuning as 2 key techniques widely used for optimizing LLM performance. The orchestration for each study could vary, and the application of specific methods depends mainly on factors including specific radiologic tasks, data size, and computational resources. Delbrouck et al. demonstrated that fine-tuning multimodal models improves the efficiency and factual accuracy of impression generation, enabling radiologists to handle higher volumes of reports without compromising quality.^55^

Likewise, combining fine-tuning with prompt optimization has proven effective when applied in modular pipelines. Soylu et al. introduce the BetterTogether algorithm to optimize language model programs by iteratively refining their weights and prompt templates. Their algorithm first optimizes prompts, then uses those optimized prompts to fine-tune the model’s weights, and finally, reoptimizes the prompts using the newly fine-tuned weights. Experimental results across diverse tasks demonstrated that this alternating strategy outperforms methods that optimize only one or the other.^56^

Privacy-preserving, open-source LLMs have great potential in various radiologic tasks and have demonstrated comparable performance with closed-source commercial models. The application of open-source models provides the opportunity for researchers to investigate model behavior and troubleshoot customized variants in addition to fine-tuning models on their specific radiology domain.^57^^,^^58^ Fine-tuned open-source models like BiomedGPT and RadFM democratize access to cutting-edge AI. These models enable smaller health care institutions to benefit from advanced AI solutions by reducing computational and financial barriers. The health care sector is governed by stringent privacy regulations, limiting commercial LLMs’ use in clinical settings. Fine-tuning and prompt optimizing open-source models address these concerns by localizing model training and inference to secure environments. Liu et al. emphasized the importance of this approach in ensuring compliance while leveraging the full potential of advanced LLMs.^38^

However, integrating these models into the radiology workflow raises several important considerations. The temperature hyperparameter that governs the randomness and creativity of the LLMs’ response is not clearly demystified. It is understood that lower temperature leads to more deterministic and conservative results, and higher temperatures generate more diverse and creative output for the same input prompt.^1^ Mukherjee et al. demonstrated that reducing an LLM’s temperature resulted in enhanced agreement for the extraction of findings from radiology reports.^59^ On the other hand, Suh et al.^60^ reported that increasing temperature settings improved the accuracy of their LLM to provide differential diagnosis from rare input images of *Diagnosis Please* cases from the *Radiology* journal. Therefore, further exploration and tuning are necessary to obtain the ideal temperature for optimal LLM application in specific radiology use cases.

Additionally, the field lacks a standardized framework for evaluating LLM outputs in radiology. While common metrics such as BLEU, ROUGE, and radiology-specific scores such as RadGraph F1, RadCliQ, and MRScore,^61^^,^^62^ have been proposed, no consensus has emerged. Given LLMs’ susceptibility to hallucinations and factual errors, ensuring transparency, interpretability, and reproducibility is critical. Explainability within the realm of large language models denotes the capacity to render the model’s reasoning processes transparent and comprehensive. Two prominent frameworks for enhancing explainability in LLMs are LIME (Local Interpretable Model-agnostic Explanations) and SHAP (SHapley Additive exPlanations). LIME identifies input features that influence a specific prediction, whereas SHAP highlights both model vulnerability and feature importance for a specific outcome. However, studies have demonstrated that LLMs do not inherently deliver plausible explanations. Therefore, there is a call for further investigation and consensus on the evaluation metrics of LLMs and assessment of their trustworthiness and reliability.^63^

Moreover, LLMs depend heavily on temporal updates for training data; thus, the rapidly evolving developments and updates in medicine can lead to insufficiency of these models once they are clinically implemented.^4^^,^^61^ Another consideration is the possibility of inheriting bias through training these models. Yang et al. revealed that LLMs like GPT-3.5-turbo and GPT-4 exhibit biases by generating skewed patient backgrounds, associating diseases with demographics, and favoring specific patient groups in treatment. Additional research is necessary for characterization and subsequently mitigating biases toward equitable and reliable care delivered by LLMs.^64^

The black box nature of LLMs also introduces risks related to system failures or unpredictable behavior during clinical implementation, which may result in regulatory and safety challenges. Addressing the technical and regulatory concerns is critical to ensure patient safety across their clinical deployment.^65^

## Conclusion

In conclusion, as LLMs continue to evolve, fine-tuning and prompt optimization are emerging as building blocks for reducing hallucinations and adapting models to specific radiology tasks. Looking ahead, key priorities include the development of standardized evaluation frameworks, implementation of continuous learning capabilities, and designing effective collaborations between radiologists and LLMs. The successful integration of these frameworks will depend on interdisciplinary partnerships among radiologists, AI researchers, and vendors that thoughtfully integrate these powerful models into clinical practice while prioritizing patient safety and equitable care.

## Supplementary Material

umaf024_Supplementary_Data
