# Supplementary material for: Decoding large language models for radiology: strategies for fine-tuning and prompt engineering
Source: Radiol Adv. 2025 Jul 28;2(4):umaf024. doi: 10.1093/radadv/umaf024 (PMC12429228; doi:10.1093/radadv/umaf024)
Supplement: umaf024_Supplementary_Data [file umaf024_Supplementary_Data.zip › Supplementary-Material-for-review.pdf]

Decoding Large Language Models for Radiology: Strategies for Fine-Tuning and Prompt Engineering

In this notebook we provide a walkthrough Large Language Models (LLMs) interaction paradigms, from prompt engineering examples to parameter-efficient fine-tuning using QLoRA for domain-specific model adaptation.

To run this notebook, you'll need to go through prerequisites below to set up access to Hugging Face models.

1. Generate a Hugging Face access Token: Go to your [Hugging Face profile]() and [create new token]().
2. Request model access: Some models on Hugging Face are "gated" and require special permission before you can access them, including popular models . To access these models, visit the specific model page (https://huggingface.co/meta-llama), (e.g. [meta-llama-3.2-1B]()).
3. Install required libraries and packages as followed below:

```
!pip install transformers datasets accelerate bitsandbytes peft trl
!pip install huggingface_hub
```

4. Configure authentication using your token:

```
import os
from huggingface_hub import login

os.environ["HUGGINGFACE_HUB_TOKEN"] = "your_token_here" # Replace 'your_token_here' with your actual token#
login(token=os.environ["HUGGINGFACE_HUB_TOKEN"])
```

Now you can load the model (e.g. Llama 3.2-1B) with automatic device detection and memory optimization. It first checks for GPU availability and attempts to load the model with 4-bit quantization for efficient memory usage. The code also configures the tokenizer with proper padding tokens to ensure compatibility with text generation pipelines.

```
from transformers import AutoTokenizer, AutoModelForCausalLM, BitsAndBytesConfig
import torch


# Model we'll be using throughout this notebook
model_name = "meta-llama/Llama-3.2-1B"

print(f" Loading {model_name}")

# Check available devices
print(f" CUDA available: {torch.cuda.is_available()}")
if torch.cuda.is_available():
    print(f" GPU: {torch.cuda.get_device_name(0)}")

# Load tokenizer first
print(" Loading tokenizer...")
tokenizer = AutoTokenizer.from_pretrained(model_name)
tokenizer.pad_token = tokenizer.eos_token


# Try GPU loading with quantization, fallback to CPU if needed
try:
    if torch.cuda.is_available():
        print(" Attempting GPU loading with 4-bit quantization...")

        # Configure 4-bit quantization for memory efficiency
        bnb_config = BitsAndBytesConfig(
            load_in_4bit=True,
            bnb_4bit_quant_type="nf4",
            bnb_4bit_compute_dtype=torch.float16,
            bnb_4bit_use_double_quant=True,
        )

        model = AutoModelForCausalLM.from_pretrained(
            model_name,
            quantization_config=bnb_config,
            device_map="auto",
            torch_dtype=torch.float16,
            trust_remote_code=True
        )
```

```
        print("Model loaded with GPU + 4-bit quantization!")

    else:
        raise RuntimeError("No GPU available, using CPU fallback")

except Exception as e:
    print(f" GPU loading failed: {e}")
    print(" Falling back to CPU loading...")

    # CPU fallback - load in float32 without quantization
    model = AutoModelForCausalLM.from_pretrained(
        model_name,
        torch_dtype=torch.float32,
        device_map="cpu",
        trust_remote_code=True
    )
    print(" Model loaded on CPU!")
    print(" Note: CPU inference will be slower")

print(" Model loading complete!")
```

Role-based prompting:

As discussed in the manuscript several LLMs operate through a structured conversation framework consisting of three distinct roles that guide the model's behavior and output generation: *system role, user role, assistant role*

The text content assigned to the system and user roles guides the assistant in generating relevant responses in the user's desired format. The system role should define the identity, characteristics, and/or tone of the model while responding to the user's question or keeping the conversation. This content is followed by the user role, which provides the user's request, including necessary background information, constraints, and clear task-specific instructions. The user's request is followed by the model's output in the assistant role. These role definitions not only structure the model's input and the generated output , but they also enable the designation of a chain of thought (CoT) process. Below is a simple example of this process:

```
# ===================================================================
#  ROLE-BASED PROMTING DATA EXTRACTION CODE
# ===================================================================
model = AutoModelForCausalLM.from_pretrained(model_name)

def get_neuroradiologist_system_prompt():
    return """You are an expert neuroradiologist. Determine if there is any intracranial hemorrhage in the radiology report and classify the
Answer with Yes/No and hemorrhage type classification. Be concise."""

def get_neuroradiologist_user_prompt(report_text):
    return f"""Review the given radiology report and conclude Is there any hemorrhage in this radiology report? If yes, what types?

{report_text}

Use your expertise to identify and classify any hemorrhage findings."""

def get_neuroradiologist_assistant_prompt():
    return """**HEMORRHAGE PRESENT:** [Yes/No]
**TYPE:** [hemorrhage type if present]"""

def create_complete_prompt(radiology_report):
    system_prompt = get_neuroradiologist_system_prompt()
    user_prompt = get_neuroradiologist_user_prompt(radiology_report)
    assistant_prompt = get_neuroradiologist_assistant_prompt()

    complete_prompt = f"{system_prompt}\n\n{user_prompt}\n\n{assistant_prompt}"
    return complete_prompt

# Sample report
sample_report = """
CT HEAD WITHOUT CONTRAST:

Acute nondisplaced fracture of the left parietal calvarium. There is an overlying left parietal scalp hematoma.
Acute subarachnoid hemorrhage in the right frontal sulci as well as the right sylvian fissure. No evidence of an intraparenchymal hematoma. N
Ventricles are normal without evidence of blood products. Preserved gray-white differentiation. There is no evidence of an acute facial fract
"""

# Generate prompt for your pipeline
prompt = create_complete_prompt(sample_report)
tokenizer.pad_token = tokenizer.eos_token
if __name__ == "__main__":
    pipe = pipeline(
        "text-generation",
        model=model,
        tokenizer=tokenizer,
```

```
        max_new_tokens=200,
        temperature=0.1,
        do_sample=True
    )


    response = pipe(prompt)
    print(response[0]['generated_text'])
```

Chain of Thought Prompting

It is another typoe of prompt potimization. As mentioned it uses the same system and user prompts as the basic approach example but adds structured reasoning steps. Instead of executing directly to the answer, the model must first work through systematic analysis steps. A detailed example is shown below where the model breaks down the radiology report analysis into clear, logical steps before reaching its final conclusion.

```
# ====================================================================
#  CHAIN OF THOUGHT DATA EXTRACTION CODE
# ====================================================================

def get_chain_of_thought_system_prompt():
    return """You are an expert neuroradiologist. Analyze radiology reports for intracranial hemorrhage using step-by-step reasoning.
Think through your analysis systematically and show your reasoning process. Be concise in your final answer."""

def get_chain_of_thought_user_prompt(report_text):
    return f"""Review the given radiology report and conclude Is there any hemorrhage in this radiology report? If yes, what types?

{report_text}

Use your expertise to identify and classify any hemorrhage findings.

Think step by step:
1. What hemorrhage-related keywords do I see?
2. Is there evidence of intracranial hemorrhage?
3. If yes, what type is described?
4. What details support this classification?"""

def get_chain_of_thought_assistant_prompt():
    return """Step 1: Keywords found:
Step 2: Hemorrhage present:
Step 3: Type identified:
Step 4: Supporting evidence:

**HEMORRHAGE PRESENT:** [Yes/No]
**TYPE:** [hemorrhage type if present]"""

def create_chain_of_thought_prompt(radiology_report):
    system_prompt = get_chain_of_thought_system_prompt()
    user_prompt = get_chain_of_thought_user_prompt(radiology_report)
    assistant_prompt = get_chain_of_thought_assistant_prompt()

    complete_prompt = f"{system_prompt}\n\n{user_prompt}\n\n{assistant_prompt}"
    return complete_prompt

# Sample report
sample_report = """
CT HEAD WITHOUT CONTRAST:

Acute nondisplaced fracture of the left parietal calvarium. There is an overlying left parietal scalp hematoma.
Acute subarachnoid hemorrhage in the right frontal sulci as well as the right sylvian fissure. No evidence of an intraparenchymal hematoma. N
Ventricles are normal without evidence of blood products. Preserved gray-white differentiation. There is no evidence of an acute facial fract
"""

# Create pipeline
pipe = pipeline(
    "text-generation",
    model=model,
    tokenizer=tokenizer,
    max_new_tokens=250,  # Increased for reasoning steps
    temperature=0.1,
    do_sample=True
)

# Generate chain of thought prompt and get response
prompt = create_chain_of_thought_prompt(sample_report)
response = pipe(prompt)
print(response[0]['generated_text'])
```

```
# ===================================================================
# ANALYZE YOUR OWN REPORTS WITH REASONING
# ===================================================================

def analyze_report_with_reasoning(your_report_text):
    """Use this function to analyze your own radiology reports with step-by-step reasoning"""
    prompt = create_chain_of_thought_prompt(your_report_text)
    response = pipe(prompt)
    return response[0]['generated_text']
```

Prompt embeddings are the numerical representations that language models use to understand and process text. When a model reads a radiology report, it converts each word into high-dimensional vectors (embeddings) that capture semantic meaning, medical context, and relationships between terms.

Below, the prompt embedding version is detailed. It uses the same system, user, and assistant prompts as the basic approach but adds embedding analysis to examine how the model internally represents the radiology text.

The code provides four key metrics:

1. Mean: Indicates if embeddings are balanced (closer to zero is better)
2. Standard Deviation: Shows consistency of representation (lower values indicate more stable encoding)
3. Min/Max Range: Reveals the spread of values (symmetric ranges suggest balanced learning)

This analysis helps validate that your prompts are being processed consistently and can guide improvements in prompt design for more reliable output generation.

```
# ============================================================
# DATA EXTRACTION WITH PROMPT EMBEDDING
# ============================================================

from transformers import pipeline
import torch

def get_neuroradiologist_system_prompt():
    return """You are an expert neuroradiologist. Determine if there is any intracranial hemorrhage in the radiology report and classify the
Answer with Yes/No and hemorrhage type classification. Be concise."""

def get_neuroradiologist_user_prompt(report_text):
    return f"""Review the given radiology report and conclude Is there any hemorrhage in this radiology report? If yes, what types?

{report_text}
Use your expertise to identify and classify any hemorrhage findings."""

def get_neuroradiologist_assistant_prompt():
    return """**HEMORRHAGE PRESENT:** [Yes/No]
**TYPE:** [hemorrhage type if present]"""

def create_complete_prompt(radiology_report):
    system_prompt = get_neuroradiologist_system_prompt()
    user_prompt = get_neuroradiologist_user_prompt(radiology_report)
    assistant_prompt = get_neuroradiologist_assistant_prompt()

    complete_prompt = f"{system_prompt}\n\n{user_prompt}\n\n{assistant_prompt}"
    return complete_prompt

def get_prompt_embeddings(prompt, tokenizer, model):
    """
    Generate embeddings for the prompt using the model's tokenizer and embeddings layer
    """
    # Tokenize the prompt
    inputs = tokenizer(prompt, return_tensors="pt", truncation=True, padding=True)

    # Move to same device as model
    if hasattr(model, 'device'):
        inputs = {k: v.to(model.device) for k, v in inputs.items()}

    # Get embeddings from the model's embedding layer
    with torch.no_grad():
        if hasattr(model, 'transformer'):
            # For GPT-style models
            embeddings = model.transformer.wte(inputs['input_ids'])
        elif hasattr(model, 'model'):
            # For other transformer models
            embeddings = model.model.embed_tokens(inputs['input_ids'])
        else:
            # Fallback approach
```

```
            embeddings = model.get_input_embeddings()(inputs['input_ids'])

    return embeddings, inputs

def analyze_with_embeddings(radiology_report, tokenizer, model, pipe):
    """
    Analyze report using both embeddings and generation pipeline
    """
    # Create the complete prompt
    prompt = create_complete_prompt(radiology_report)

    # Get prompt embeddings
    embeddings, token_inputs = get_prompt_embeddings(prompt, tokenizer, model)

    # Print embedding information
    print("PROMPT EMBEDDING ANALYSIS:")
    print(f"Prompt length: {len(prompt)} characters")
    print(f"Token count: {embeddings.shape[1]} tokens")
    print(f"Embedding dimensions: {embeddings.shape[2]}")
    print(f"Embedding tensor shape: {embeddings.shape}")
    print("-" * 50)

    # Generate response using pipeline
    response = pipe(prompt)

    return response, embeddings, token_inputs

# Sample report
sample_report = """
CT HEAD WITHOUT CONTRAST:

Acute nondisplaced fracture of the left parietal calvarium. There is an overlying left parietal scalp hematoma.
Acute subarachnoid hemorrhage in the right frontal sulci as well as the right sylvian fissure. No evidence of an intraparenchymal hematoma. N
Ventricles are normal without evidence of blood products. Preserved gray-white differentiation. There is no evidence of an acute facial fract
"""

# Create pipeline
pipe = pipeline(
    "text-generation",
    model=model,
    tokenizer=tokenizer,
    max_new_tokens=50,
    temperature=0.1,
    do_sample=True
)

# Analyze with embeddings
response, embeddings, token_inputs = analyze_with_embeddings(sample_report, tokenizer, model, pipe)

print("MODEL RESPONSE:")
print(response[0]['generated_text'])
print("\nEMBEDDING STATISTICS:")
print(f"Mean embedding value: {embeddings.mean().item():.6f}")
print(f"Std embedding value: {embeddings.std().item():.6f}")
print(f"Min embedding value: {embeddings.min().item():.6f}")
print(f"Max embedding value: {embeddings.max().item():.6f}")

def analyze_report_with_embeddings(your_report_text):
    """Use this function to analyze your own radiology reports with embedding analysis"""
    response, embeddings, token_inputs = analyze_with_embeddings(your_report_text, tokenizer, model, pipe)
    return response[0]['generated_text'], embeddings
```

QLoRA Fine-Tuning Setup

QLoRA enables efficient *fine-tuning* of large language models using significantly less computational resources while maintaining performance quality. The example below provides a pipeline from interactive dataset upload (train/validation/test set files) through optimized training with memory constraints, to comprehensive evaluation and inference testing on new radiology reports.

The code includes configuration parameters for LoRA (rank, alpha, dropout), training hyperparameters (learning rate, batch size, epochs), and 4-bit quantization settings to optimize memory usage. All key QLoRA parameters including LoRA rank, quantization settings, learning rate, and training configurations are centralized in a CONFIG dictionary for easy customization and experimentation without modifying multiple functions throughout the code.

To apply this code you should upload your datasets to begin fine-tuning your model for specialized medical text analysis with functions that handle model loading with QLoRA configuration, dataset preparation, training execution, performance evaluation, and inference testing on new reports.

```
# ====================================================================
# INSTALL PACKAGES & IMPORT LIBRARIES
# ====================================================================

!pip install transformers datasets peft accelerate bitsandbytes trl pandas scikit-learn

import torch
from transformers import (
    AutoTokenizer,
    AutoModelForCausalLM,
    BitsAndBytesConfig,
    TrainingArguments,
    pipeline
)
from peft import LoraConfig, get_peft_model, TaskType, prepare_model_for_kbit_training
from datasets import Dataset, load_dataset
from trl import SFTTrainer
import pandas as pd
import json
from sklearn.metrics import accuracy_score, classification_report

# ====================================================================
# CONFIGURATION - ADJUST THESE SETTINGS FOR YOUR NEEDS
# ====================================================================

CONFIG = {
    # LoRA Parameters
    "lora_r": 16,  # Rank (8, 16, 32, 64) - higher = more parameters
    "lora_alpha": 32,  # Scaling (usually 2x rank)
    "lora_dropout": 0.1,  # Dropout (0.05-0.1)

    # Training Parameters
    "learning_rate": 2e-4,
    "num_epochs": 2,
    "batch_size": 1,
    "gradient_accumulation": 4,  # Effective batch size multiplier
    "max_seq_length": 512,  # Max sequence length (256-1024)
    "max_steps": 200,

    # Evaluation Parameters
    "eval_steps": 50,  # How often to evaluate
    "save_steps": 100,  # How often to save
    "warmup_steps": 50,

    # Generation Parameters
    "max_new_tokens": 50,
    "temperature": 0.1
    }

# ====================================================================
# QLORA CONFIGURATION
# ====================================================================

def get_qlora_config():
    """Configure QLoRA with 4-bit quantization"""
    bnb_config = BitsAndBytesConfig(
        load_in_4bit=True,
        bnb_4bit_use_double_quant=True,
        bnb_4bit_quant_type="nf4",
        bnb_4bit_compute_dtype=torch.float16
    )
    return bnb_config

def get_lora_config():
    """Configure LoRA parameters for medical text"""
    lora_config = LoraConfig(
        task_type=TaskType.CAUSAL_LM,
        inference_mode=False,
        r=CONFIG["lora_r"],
        lora_alpha=CONFIG["lora_alpha"],
        lora_dropout=CONFIG["lora_dropout"],
        target_modules=["q_proj", "v_proj", "k_proj", "o_proj"]
    )
    return lora_config

def load_model_with_qlora(model_name):
    """Load model with QLoRA configuration"""
    bnb_config = get_qlora_config()

    tokenizer = AutoTokenizer.from_pretrained(model_name)
    if tokenizer.pad_token is None:
        tokenizer.pad_token = tokenizer.eos_token
```

```python
    model = AutoModelForCausalLM.from_pretrained(
        model_name,
        quantization_config=bnb_config,
        device_map="auto",
        torch_dtype=torch.float16
    )

    model = prepare_model_for_kbit_training(model)
    lora_config = get_lora_config()
    model = get_peft_model(model, lora_config)

    return model, tokenizer

# ====================================================================
# LOAD YOUR MODEL
# ====================================================================

# REPLACE WITH YOUR MODEL NAME
MODEL_NAME = "meta-llama/Llama-3.2-1B"

print("Loading model with QLoRA configuration...")
model, tokenizer = load_model_with_qlora(MODEL_NAME)
print("Model loaded successfully!")

# ====================================================================
# UPLOAD YOUR DATASETS
# ====================================================================

# Upload your train, validation, and test CSV files to Colab
# Expected columns: 'report', 'hemorrhage_present', 'hemorrhage_type'

from google.colab import files

print("Upload your training dataset (CSV format):")
uploaded_train = files.upload()
train_file = list(uploaded_train.keys())[0]

print("Upload your validation dataset (CSV format):")
uploaded_val = files.upload()
val_file = list(uploaded_val.keys())[0]

print("Upload your test dataset (CSV format):")
uploaded_test = files.upload()
test_file = list(uploaded_test.keys())[0]

print(f"Files uploaded: {train_file}, {val_file}, {test_file}")

# ====================================================================
#  DATASET PREPARATION FUNCTIONS
# ====================================================================

def load_datasets(train_path, val_path, test_path):
    """Load train, validation, and test datasets"""
    print("Loading datasets...")

    train_df = pd.read_csv(train_path)
    val_df = pd.read_csv(val_path)
    test_df = pd.read_csv(test_path)

    print(f"Training samples: {len(train_df)}")
    print(f"Validation samples: {len(val_df)}")
    print(f"Test samples: {len(test_df)}")

    # Show sample data
    print("\nSample training data:")
    print(train_df.head(2))

    return train_df, val_df, test_df

def create_training_prompt(report, hemorrhage_present, hemorrhage_type):
    """Create formatted training prompt"""
    # Create the answer
    if hemorrhage_present:
        answer = f"**HEMORRHAGE PRESENT:** Yes\n**TYPE:** {hemorrhage_type}"
    else:
        answer = f"**HEMORRHAGE PRESENT:** No\n**TYPE:** None"

    # Simple format: question + report + answer
    full_prompt = f"Is there any hemorrhage in this radiology report? If yes, what type?\n\n{report}\n\n{answer}"
    return full_prompt

def prepare_dataset_for_training(df):
    """Convert dataframe to training format"""
```

```
    training_texts = []
    for _, row in df.iterrows():
        prompt = create_training_prompt(
            row['report'],
            row['hemorrhage_present'],
            row['hemorrhage_type']
        )
        training_texts.append({"text": prompt})

    return Dataset.from_list(training_texts)

# Load and prepare datasets
train_df, val_df, test_df = load_datasets(train_file, val_file, test_file)
train_dataset = prepare_dataset_for_training(train_df)
val_dataset = prepare_dataset_for_training(val_df)

print("Datasets prepared for training!")

# ====================================================================
# TRAINING FUNCTION
# ====================================================================

def train_qlora_model(model, tokenizer, train_dataset, val_dataset):
    """Fine-tune model with QLoRA using train and validation sets"""

    training_args = TrainingArguments(
        output_dir="./ich-qlora-model",
        per_device_train_batch_size=CONFIG["batch_size"],
        per_device_eval_batch_size=CONFIG["batch_size"],
        gradient_accumulation_steps=CONFIG["gradient_accumulation"],
        warmup_steps=CONFIG["warmup_steps"],
        num_train_epochs=CONFIG["num_epochs"],
        learning_rate=CONFIG["learning_rate"],
        fp16=True,
        logging_steps=10,
        eval_steps=CONFIG["eval_steps"],
        save_steps=CONFIG["save_steps"],
        evaluation_strategy="steps",
        save_strategy="steps",
        load_best_model_at_end=True,
        metric_for_best_model="eval_loss",
        greater_is_better=False,
        report_to=None,
        group_by_length=True,
        lr_scheduler_type="cosine",
        warmup_ratio=0.05,
        max_steps=CONFIG["max_steps"],
    )

    trainer = SFTTrainer(
        model=model,
        train_dataset=train_dataset,
        eval_dataset=val_dataset,
        args=training_args,
        tokenizer=tokenizer,
        max_seq_length=CONFIG["max_seq_length"],
        dataset_text_field="text",
        packing=False,
    )

    print("Starting QLoRA fine-tuning...")
    trainer.train()

    trainer.save_model()
    print("Training completed! Model saved!")

    return trainer

# ====================================================================
# START TRAINING
# ====================================================================

# Start the training process
trainer = train_qlora_model(model, tokenizer, train_dataset, val_dataset)

print(" Training completed successfully!")

# ====================================================================
# EVALUATION FUNCTIONS
# ====================================================================

def create_inference_prompt(report):
    """Create prompt for inference (without answer)"""
```

```python
    prompt = f"Is there any hemorrhage in this radiology report? If yes, what type?\n\n{report}\n\n"
    return prompt

def evaluate_model(model, tokenizer, test_df):
    """Evaluate fine-tuned model on test dataset"""
    print("Evaluating model on test dataset...")

    # Create inference pipeline
    pipe = pipeline(
        "text-generation",
        model=model,
        tokenizer=tokenizer,
        max_new_tokens=CONFIG["max_new_tokens"],
        temperature=CONFIG["temperature"],
        do_sample=True,
        pad_token_id=tokenizer.eos_token_id
    )

    predictions = []
    true_labels = []

    for i, row in test_df.iterrows():
        # Create inference prompt
        prompt = create_inference_prompt(row['report'])

        # Generate prediction
        response = pipe(prompt)
        generated_text = response[0]['generated_text']

        # Extract prediction
        if "HEMORRHAGE PRESENT:** Yes" in generated_text:
            predicted_hemorrhage = True
        else:
            predicted_hemorrhage = False

        predictions.append(predicted_hemorrhage)
        true_labels.append(row['hemorrhage_present'])

        # Print first 3 examples
        if i < 3:
            print(f"\nExample {i+1}:")
            print(f"Report: {row['report'][:100]}...")
            print(f"True: {row['hemorrhage_present']}, Predicted: {predicted_hemorrhage}")
            print(f"Generated: {generated_text.split(prompt)[-1][:100]}...")

    # Calculate metrics
    accuracy = accuracy_score(true_labels, predictions)
    report = classification_report(true_labels, predictions)

    print(f"\n Test Accuracy: {accuracy:.4f}")
    print("\n Classification Report:")
    print(report)

    return accuracy, predictions, true_labels

# ===================================================================
# EVALUATE MODEL
# ===================================================================

# Evaluate the fine-tuned model
accuracy, predictions, true_labels = evaluate_model(model, tokenizer, test_df)

# ===================================================================
# TEST WITH NEW REPORTS
# ===================================================================

def analyze_new_report(report_text, model, tokenizer):
    """Analyze a new radiology report with the fine-tuned model"""
    pipe = pipeline(
        "text-generation",
        model=model,
        tokenizer=tokenizer,
        max_new_tokens=CONFIG["max_new_tokens"],
        temperature=CONFIG["temperature"],
        do_sample=True,
        pad_token_id=tokenizer.eos_token_id
    )

    prompt = create_inference_prompt(report_text)
    response = pipe(prompt)

    return response[0]['generated_text']
```

```
# Test with a sample report
test_report = """
CT HEAD WITHOUT CONTRAST:
Large acute intraparenchymal hemorrhage in the right basal ganglia measuring 4.2 x 3.1 x 3.5 cm
with surrounding vasogenic edema. Mass effect is present with 8 mm leftward midline shift.
"""

result = analyze_new_report(test_report, model, tokenizer)
print(" New Report Analysis:")
print(result)

# ===================================================================
# SAVE MODEL TO DRIVE (OPTIONAL)
# ===================================================================

# Optional: Save to Google Drive
from google.colab import drive
drive.mount('/content/drive')
```

Thank you for following along with this notebook.
